# Removal of Superior Maxilla and Preservation of Facial Expression

**Published:** 1900-03

**Authors:** C. B. Parker

**Affiliations:** New York


### REMOVAL OF SUPERIOR MAXILLA AND PRESERVATION OF FACIAL EXPRESSION.[1]

BY DR. C. B. PARKER, NEW YORK.

I AM not in the habit of apologizing, but I think I ought to, when you hear how little I have to say. I do not intend to read a scientific paper and go into detailism, but more especially to speak of facts and methods where results have been very satisfactory in my work. I shall be as brief as possible.

I do not think it is necessary to describe in detail the operation for the removal of the superior maxilla or a portion of it, before this Society, but to speak more particularly concerning the preservation of the natural facial expression after the operation. Assuming that an operation is necessary, the anæsthetic to be used is the next important thought to be considered. I much prefer the use of the local to the general anæsthetic in this class of cases, for the patient often gives me aid and comfort in my work. In nine out of ten cases, I prefer and do use beta-eucain; generally, a two per cent. solution I find of sufficient strength. In fact, for seventy-five per cent. of all ordinary operations, I think it more satisfactory than anything I know of to-day.

By using two per cent. of eucain hypodermically, and waiting fifteen minutes, the anæsthesia or analgesia is complete. This preparation is safer than cocaine, notwithstanding the assurance given us that volasem is the antidote to counteract its physiological action.

For all operations on the maxillary bones (as I confine myself to them), I do not find use for the bone-forceps or saw, especially in the upper jaw. I depend on the surgical engine and the instruments used with it, such as the trephine, drills, burs, and occasionally the bone-curette.

Seldom is it necessary to use the knife or disfigure the face for

---

[1] Read before the New York Institute of Stomatology, November 9, 1899.

the removal of the maxillary bones, when one has been educated in the use of the surgical engine. With the aid of the periostotome, the soft tissues are separated from the bone and held out of the way, making the operation comparatively bloodless. Then with the engine and fissure drill, the narcotic parts, such as in necrosis, are cut around and dislodged by the periostotome, as an elevator, or the slender root-forcep, that we are all familiar with. In case of caries, to cut around the supposed zone, and if not complete, then with the round bur follow it up till all the softened bone is removed.

The great advantage in this method of operating is, that you can cut ahead of you, and not destroy laterally any more bony tissue than is necessary, nor scar the face, as is very often done to make room where the bone-forceps and saw are to be used.

Furthermore, what I consider an important feature in this work is that there are clean, smooth edges of the bone, and with very little loss of blood. We have now reached a very important part of this subject, and which may be of especial interest to the dentist, that is, preserving the facial expression, which is so often destroyed by the removal of the superior maxilla or a large portion of it. Since I was asked to speak on this subject, I have been able to obtain a photograph, and consent to use it for this meeting to-night, which tells its own story, as the picture was taken with the artificial denture removed from the mouth. I am glad to show this picture and the result, as it is a patient I operated for last winter, while two members of this Institute were present and will no doubt recognize the person. It is not necessary here to give the history; further, that after suffering much pain for about three months, his physician referred him to me for the removal of whatever necrotic bone was necessary from the superior maxilla. Caries was easily diagnosed, and I removed from the molar of the right side, the alveolus, a portion of malar and frontal processes extending across to the left side (as shown in the picture), taking away the frontal, malar, and alveolar process to the second molar, as well as the palatine process on both sides, leaving only the posterior third.

It can be readily seen that most of the bones that fix and support the anterior facial expression were removed, and the usual result of such an operation is anything but pleasing to the eye in after times, by the sinking in of the nose and features in general.

A method of preserving the natural facial expression in this class of cases, whether the operation has been large or small, is by the use of sheet-lead, so shaped as to restore, lift up, or hold out the face in its natural position, retaining it there until recovery is so nearly completed that it is of no further use or cannot be retained longer. The lead having been fitted in place, it is then removed and wrapped with at least one layer of surgical gauze, preferably boric acid, being careful to have the ends well covered if it is resisting much force, as the thin edges might irritate the soft tissues. It is then placed in position, and with the fingers it is molded in its proper shape, the metal being soft and pliable enough for that purpose. The cavity is then packed with surgical gauze underneath the lead shield, being careful never to pack to the extreme bottom of the cavity, for in so doing the new tissue is held in restraint and cicatrix formed.

As granulation takes place, the lead must be removed and made narrower, to prevent the new tissue that is forming from coming in contact with it in the bottom of the cavity.

Repeat the cutting away of the lead shield from time to time, until the cavity is nearly or completely filled with the new fibrous or cellular tissue.

